# Supplementary material for: Association of the SH2B1 rs7359397 Gene Polymorphism with Steatosis Severity in Subjects with Obesity and Non-Alcoholic Fatty Liver Disease
Source: Nutrients. 2020 Apr 29;12(5):1260. doi: 10.3390/nu12051260 (PMC7282006; doi:10.3390/nu12051260)
Supplement: Supplementary file 1 [file nutrients-12-01260-s001.pdf]

Table S1. Logistic regression analysis between Fatty Liver Index and genotype in NAFLD subjects.

	OR (95% CI)	p-value
**rs7359397 *SH2B1***		
CC (No risk genotype)	Reference	
CT/TT (Risk genotype)	2.91 (1.07;7.91)	0.036
Sex	0.22 (0.07;0.64)	0.006
Age	0.94 (0.89;1.00)	0.060
ALT	3.72 (1.29;10.7)	0.015
Physical activity	0.88 (0.55;1.40)	0.602
Total energy intake	1.00 (0.99;1.00)	0.186
	**Pseudo R^2 0.2472**	**p-model <0.001**

p<0.05 was considered statistically significance. Odds Ratio (95% confidence interval) for hepatic steatosis were compared by logistic regression. ALT, Alanine aminotransferase. FLI was categorized according to the median (<80 vs. ≥80).

Table S2. Logistic regression analysis showing the association between steatosis degree measured by ultrasonography and genotype risk in NAFLD subjects.

	OR (95% CI)	p-value
**rs7359397 *SH2B1***		
CC (No risk genotype)	Reference	
CT/TT (Risk genotype)	4.15 (1.59;10.8)	0.004
Sex	0.97 (0.34;2.76)	0.956
Age	1.03 (0.98;1.09)	0.183
BMI	1.16 (1.01;1.32)	0.027
ALT	7.01 (2.03;21.37)	<0.001
Physical activity	0.88 (0.57;1.36)	0.567
Adherence to MedDiet	0.99 (0.78;1.25)	0.935
	**Pseudo R^2 0.2434**	**p-model <0.001**

p<0.05 was considered statistically significance. Odds Ratio (95% confidence interval) for hepatic steatosis were compared by logistic regression. ALT, Alanine aminotransferase; BMI, Body Mass Index.

Table S3. Multinomial logistic regression model with *SH2B1* genotype as independent variable and Liver fat content assessed by Magnetic Resonance Imaging (MRI) as dependent variable.

	**RRR (95% CI)**	**p-value**
**MRI Liver fat content**		
**T1 (0.2 – 4.5%)**	Reference	
**T2 (4.6 – 8.9%)**		
CC (No-risk genotype)	Reference	
CT/TT (Risk genotype)	1.68 (0.58;4.88)	0.337
Sex	0.42 (0.11;1.58)	0.202
Age	0.98 (0.92;1.02)	0.649
BMI	1.03 (0.89;1.20)	0.635
ALT	1.64 (0.40;6.72)	0.490
Physical activity	0.52 (0.30; 0.90)	0.021
Adherence to MedDiet	1.10 (0.84;1.42)	0.470
**T3 (9.3 – 62.5%)**		
CC (No-risk genotype)	Reference	
CT/TT (Risk genotype)	3.93 (1.09;14.10)	0.036
Sex	1.04 (0.27;4.02)	0.948
Age	1.03 (0.96;1.10)	0.308
BMI	1.08 (0.93;1.27)	0.284
ALT	36.6 (7.03;191.22)	<0.001
Physical activity	0.76 (0.43;1.33)	0.346
Adherence to MedDiet	0.84 (0.60;1.17)	0.309
	**Pseudo R^2 0.2321**	**p-model <0.001**

Relative Risk Ratio (95% confidence interval) for Liver fat content assessed by Magnetic Resonance Imaging (MRI) were compared by multinomial logistic regression. $p<0.05$ was considered statistically significance. ALT, Alanine aminotransferase; BMI, Body Mass Index; MRI, Magnetic Resonance Imaging.

Table S4. Multinomial logistic regression model with *SH2B1* genetic variant as an independent variable and the diagnostic of steatosis or steatohepatitis (by lipidomic analysis) as dependent variable.

	**RRR (95% CI)**	**p-value**
**OWLiver®-Test**		
No NAFLD or inconclusive	Reference	
Hepatic steatosis		
CC (No-risk genotype)	Reference	
CT/TT (Risk genotype)	4.77 (1.13;20.09)	0.033
Sex	3.20 (0.70;14.64)	0.132
Age	1.08 (1.00;1.18)	0.047
ALT	2.61 (0.62;10.98)	0.191
Physical activity	0.64 (0.35;1.14)	0.131
Adherence to MedDiet	0.83 (0.59; 1.18)	0.313
NASH		
CC (No-risk genotype)	Reference	
CT/TT (Risk genotype)	7.88 (2.08;29.75)	0.002
Sex	2.76 (0.65;11.6)	0.164
Age	1.04 (0.96;1.12)	0.282
ALT	2.55 (0.68;9.55)	0.165
Physical activity	0.53 (0.31; 0.90)	0.020
Adherence to MedDiet	1.05 (0.78; 1.43)	0.712
	**Pseudo R^2 0.1211**	**p-model <0.013**

Relative Risk Ratio (95% confidence interval) for the diagnostic of steatosis or steatohepatitis (by lipidomic analysis) by multinomial logistic regression. p<0.05 was considered statistically significance. ALT, Alanine aminotransferase; NAFLD: Non-alcoholic Fatty Liver Disease; NASH, Non-alcoholic steatohepatitis.
